# Caveolin 3‐dependent loss of t‐tubular *I*_Ca_ during hypertrophy and heart failure in mice

**DOI:** 10.1113/EP086731

**Published:** 2018-04-14

**Authors:** Simon M. Bryant, Cherrie H. T. Kong, Judy J. Watson, Hanne C. Gadeberg, Andrew F. James, Mark B. Cannell, Clive H. Orchard

**Affiliations:** ^1^ School of Physiology, Pharmacology and Neuroscience University of Bristol Bristol UK

**Keywords:** calcium current, caveolin 3, heart failure, t‐tubules

## Abstract

**New Findings:**

- **What is the central question of this study?**
  Heart failure is associated with redistribution of L‐type Ca^2+^ current (*I*_Ca_) away from the t‐tubule membrane to the surface membrane of cardiac ventricular myocytes. However, the underlying mechanism and its dependence on severity of pathology (hypertrophy *versus* failure) are unclear.
- **What is the main finding and its importance?**
  Increasing severity of response to transverse aortic constriction, from hypertrophy to failure, was accompanied by graded loss of t‐tubular *I*_Ca_ and loss of regulation of *I*_Ca_ by caveolin 3. Thus, the pathological loss of t‐tubular *I*_Ca_, which contributes to impaired excitation–contraction coupling and thereby cardiac function *in vivo*, appears to be attributable to loss of caveolin 3‐dependent stimulation of t‐tubular *I*_Ca_.

**Abstract:**

Previous work has shown redistribution of L‐type Ca^2+^ current (*I*_Ca_) from the t‐tubules to the surface membrane of rat ventricular myocytes after myocardial infarction. However, whether this occurs in all species and in response to other insults, the relationship of this redistribution to the severity of the pathology, and the underlying mechanism, are unknown. We have therefore investigated the response of mouse hearts and myocytes to pressure overload induced by transverse aortic constriction (TAC). Male C57BL/6 mice underwent TAC or equivalent sham operation 8 weeks before use. *I*_Ca_ and Ca^2+^ transients were measured in isolated myocytes, and expression of caveolin 3 (Cav3), junctophilin 2 (Jph2) and bridging integrator 1 (Bin1) was determined. C3SD peptide was used to disrupt Cav3 binding to its protein partners. Some animals showed cardiac hypertrophy in response to TAC with little evidence of heart failure, whereas others showed greater hypertrophy and pulmonary congestion. These graded changes were accompanied by graded cellular hypertrophy, t‐tubule disruption, decreased expression of Jph2 and Cav3, and decreased t‐tubular *I*_Ca_ density, with no change at the cell surface, and graded impairment of Ca^2+^ release at t‐tubules. C3SD decreased *I*_Ca_ density in control but not in TAC myocytes. These data suggest that the graded changes in cardiac function and size that occur in response to TAC are paralleled by graded changes in cell structure and function, which will contribute to the impaired function observed *in vivo*. They also suggest that loss of t‐tubular *I*_Ca_ is attributable to loss of Cav3‐dependent stimulation of *I*_Ca_.

## 1 INTRODUCTION

Contraction of cardiac ventricular myocytes is initiated by Ca^2+^ influx across the cell membrane during the action potential (AP), via L‐type Ca^2+^ channels (generating the L‐type Ca^2+^ current, *I*_Ca_; for review see Bers, 2002). This triggers Ca^2+^‐induced Ca^2+^ release (CICR) from adjacent sarcoplasmic reticulum (SR), which produces the Ca^2+^ transient that activates contraction. Both *I*_Ca_ and CICR occur predominantly at the t‐tubules, which are invaginations of the surface membrane that ensure synchronous Ca^2+^ release throughout the cell (Cheng, Cannell, & Lederer, 1994; Kawai, Hussain, & Orchard, 1999) and are therefore central to excitation–contraction (E–C) coupling.

During heart failure, Ca^2+^ release from the SR is compromised (Bers, 2006; Gómez et al., 1997a), although most studies show little change of *I*_Ca_ (Benitah et al., 2002; Mukherjee & Spinale, 1998; Richard et al., 1998). However, recent work has shown that heart failure induced in rats by coronary artery ligation (CAL) is associated with a decrease in *I*_Ca_ density at the t‐tubules and a compensatory increase at the cell surface, which results in a more uniform spatial distribution of *I*_Ca_ and little overall change in *I*_Ca_ density (Bryant et al., 2015); similar changes have been reported in failing human hearts (Sanchez‐Alonso et al., 2016). The more uniform distribution might help to explain the paradoxical lack of change in whole‐cell *I*_Ca_ density despite the loss of t‐tubules reported in previous studies (Balijepalli et al., 2003; Crossman, Ruygrok, Soeller, & Cannell, 2011; He et al., 2001; Lyon et al., 2009; Song et al., 2006; Wei et al., 2010). Nevertheless, the loss of *I*_Ca_ at the t‐tubules is associated with impaired local Ca^2+^ release (Bryant et al., 2015), which will contribute to the smaller slower systolic Ca^2+^ transient, and thus contraction, observed in heart failure (Frisk et al., 2016; Louch et al., 2004, 2006).

However, it is unclear how these changes are related to the degree of pathology, in particular the transition between hypertrophy and overt failure, although it has recently been suggested that classification of failing hearts might, in future, be based ‘less on measures of overall organ function such as ejection fraction, and more on the molecular details of myocyte, and in particular t‐tubule, pathophysiology’ (Hong & Shaw, 2017). The cause of the redistribution of *I*_Ca_ is also unknown, although the scaffolding protein caveolin 3 (Cav3) might play a role (Bryant et al., 2015; Bryant, Kong, Cannell, Orchard, & James, 2018), because it appears to localize *I*_Ca_ to the t‐tubules (Bryant et al., 2014) and undergoes downregulation in heart failure (Feiner et al., 2011; Fridolfsson & Patel, 2013). We have, therefore, performed a *post hoc* analysis of the response to transverse aortic constriction (TAC) in mice, to investigate the relationship between the whole‐heart and cellular responses to TAC, and investigated the effect of decreasing protein binding to Cav3 on *I*_Ca_ in ventricular myocytes from normal and TAC hearts.

## 2 METHODS

### 2.1 Ethical approval

All animal procedures were approved by the Animal Welfare and Ethics Review Board of the University of Bristol (14/6/2016) and conducted in accordance with UK legislation [Animals (Scientific Procedures) Act 1986 Amendment Regulations 2012 incorporating European Directive 2010/63/EU]; the study also complies with the ethical principles under which *Experimental Physiology* operates.

Data from 34 male wild‐type (WT) C57Bl/6 mice, specifically bred at the University of Bristol, are reported in this study; surgery was performed at 12 weeks of age and myocyte isolation at 20 weeks of age. Mice were kept in a temperature‐controlled, enriched environment with *ad libitum* access to food and water.

Transverse aortic constriction was used to produce pressure overload, which results in cardiac hypertrophy and heart failure (Rockman et al., 1991; Tachibana, Naga Prasad, Lefkowitz, Koch, & Rockman, 2005). Briefly, animals were anaesthetized with ketamine (75 mg kg^−1^ i.p., Zoetis UK Ltd., London, UK) and medetomidine (1 mg kg^−1^ i.p., Orion Corporation, Espoo, Finland) and given buprenorphine (0.05 mg kg^−1^ s.c., Reckitt Benckiser Health Care (UK) Ltd., Hull, North Humberside, UK) for pain relief; the surgical plane of anaesthesia was monitored using the limb withdrawal reflex. The aortic arch was exposed via a medial sternal thoracotomy and a silk ligature (6–0) placed between the innominate and left carotid arteries and tied round a 27‐gauge needle (0.41 mm o.d.). Sham animals underwent the same operation but without placement of the banding suture. Animals were maintained postoperatively for 8 weeks before use. There was no significant difference between sham‐operated and age‐matched control animals for any of the variables measured, so these data were pooled to form the control group. The ratio of lung weight to tibia length (LW:TL) was taken as an index of heart failure and used to separate the TAC animals into two groups: hypertrophy (HTY) and heart failure (HF; see Results).

*In vivo* cardiac function was monitored using echocardiography. Animals were anaesthetized (isoflurane 1–3%, Merial Animal Health Ltd., Harlow, Essex, UK), heart rate was monitored, and measurements of contraction were made from M‐mode images acquired from the parasternal short‐axis view using a Vevo 3100 (FUJIFILM VisualSonics Inc., Toronto, Ontario, Canada) and an MX550D transducer.

### 2.2 Myocyte isolation and detubulation

Animals were injected with heparin (500 I.U., i.p.) and killed by cervical dislocation. The heart was rapidly excised and Langendorff perfused at 37°C, and myocytes were isolated using a standard enzymatic method (Bryant et al., 2014). Myocyte detubulation (DT; physical and functional uncoupling of the t‐tubules from the surface membrane) was achieved using formamide‐induced osmotic shock, as described previously (Brette & Orchard, 2003; Bryant et al., 2014, 2015; Kawai et al., 1999); this procedure has no effect on *I*_Ca_, the action potential or the systolic Ca^2+^ transient in rat atrial cells, which lack t‐tubules (Brette, Komukai, & Orchard, 2002).

### 2.3 Solutions

Cells were superfused with a solution that contained (in mmol l^−1^): 133 NaCl, 5 KCl, 1 MgSO_4_, 1 CaCl_2_, 1 Na_2_HPO_4_, 10 d‐glucose and 10 Hepes (pH adjusted to 7.4 with NaOH). During electrophysiological recordings, KCl was replaced with CsCl to inhibit K^+^ currents. The pipette solution contained (in mmol l^−1^): 110 CsCl, 20 TEACl, 0.5 MgCl_2_, 5 MgATP, 5 BAPTA, 10 Hepes and 0.4 GTP‐Tris (pH adjusted to 7.2 with CsOH). All experiments were performed at room temperature.

C3SD peptide (Pepceuticals Ltd., Enderby, Leicestershire, UK) was used to disrupt binding of Cav3 to its protein partners (Bryant et al., 2014; Couet, Li, Okamoto, Ikezu, & Lisanti, 1997; Feron et al., 1998; MacDougall et al., 2012). Myocytes were incubated in 1 μmol l^−1^ TAT‐C3SD for 45 min at room temperature before use; we have previously shown that a scrambled version of this peptide has no effect on *I*_Ca_ (Bryant et al., 2014). C3SD corresponds to the scaffolding domain of Cav3; it was designed to compete with endogenous Cav3 for its protein partners (Couet et al., 1997) and has previously been shown to inhibit Cav3‐dependent signalling (Feron et al., 1998; MacDougall et al., 2012).

### 2.4 Recording and analysis of *I*_Ca_

Myocytes were placed in a chamber mounted on a Nikon Diaphot inverted microscope. Membrane currents and cell capacitance were recorded using the whole‐cell patch‐clamp technique, using an Axopatch 200B, Digidata 1322A A/D converter and pClamp 10 (Molecular Devices, LLC. San Jose, CA, United States). Pipette resistance was typically 1.5–3 MΩ when filled with pipette solution, and pipette capacitance and series resistance were compensated by ∼70%. Currents were activated from a holding potential of −80 mV by a step depolarization to −40 mV for 200 ms (to inactivate the sodium current, *I*_Na_) followed by steps to potentials between −50 and +80 mV for 500 ms, before repolarization to the holding potential, at a frequency of 0.2 Hz. The amplitude of *I*_Ca_ (in picoamperes) was measured as the difference between the peak inward current and the current at the end of the depolarizing pulse, and was normalized to cell capacitance (in picofarads; a function of membrane area) to calculate *I*_Ca_ density (in picoamperes per picofarad). The inactivation phase of *I*_Ca_, during the step depolarization, was fitted to a double exponential function to give fast (τ_f_) and slow (τ_s_) time constants. Surface membrane current density was obtained from currents measured in DT myocytes after correction for incomplete DT, as described previously (Bryant et al., 2014, 2015; Horiuchi‐Hirose et al., 2011; Kawai et al., 1999); t‐tubular membrane current density was calculated from the loss of membrane current and capacitance after DT. Detubulation efficiency, measured from images of intact and DT cells stained with di‐8‐ANEPPS, was ∼91%, and was not different between the groups.

### 2.5 Confocal imaging and analysis of cell membrane

The surface and t‐tubular cell membranes were stained by incubating cells with 5 μmol l^−1^ di‐8‐ANEPPS for 10 min. Volume imaging of labelled cells was carried out using an LSM 880 confocal microscope (Zeiss, Carl Zeiss AG, Oberkochen, Germany) in Airyscan ‘super‐resolution’ mode, with a 1.4 numerical aperture (NA) ×63 oil immersion objective, with voxel size set to 40 nm in plane and 180 nm along the optical axis. Image analysis was performed using MATLAB R2015a (Mathworks Inc., Natick, MA, USA) and ImageJ (v1.50; National Institutes of Health, Bethesda, MD, USA). The regularity of t‐tubule staining was quantified by applying a two‐dimensional (2D) fast Fourier transformation to an offset‐subtracted square region of the interior of the cell, and the power of the first harmonic normalized to that of the average image intensity (*P*_1_/*P*_0_). The t‐tubule density and orientation were obtained by first processing the volumetric data with a tubule‐enhancing three‐dimensional (3D) filter, segmenting using an Otsu threshold [graythresh(), Matlab, Mathworks Inc.] and skeletonizing using ‘Skeletonize (2D/3D)’ in ImageJ. The skeleton was then used to calculate t‐tubule density (length per cell volume, in micrometres per cubic micrometre) and as a mask to locate Eigenvectors for extracting t‐tubule angles. Tubule orientation is expressed relative to the z‐disc plane, so that 0 deg corresponds to a transverse tubule, whereas 90 deg corresponds to a tubule that is orthogonal to the z‐disc.

### 2.6 Confocal measurement of intracellular Ca^2+^ and membrane potential

Cells were imaged using a water immersion objective with 1.2 NA and ×40 magnification, with the confocal pinhole set to 1 Airy unit. Line‐scans were recorded at 0.5–1.0 ms per line. Cells were field stimulated at 0.2 Hz at 1.5 × threshold using parallel Pt wires.

Intracellular Ca^2+^ and membrane potential were recorded simultaneously at a single t‐tubule in myocytes loaded with the Ca^2+^ indicator fluo‐4 AM (5 μmol l^−1^ for 25 min; Thermo Fisher Scientific, Waltham, MA, USA) and the voltage‐sensitive dye di‐4‐AN(F)EPPTEA (0.5–1 μg ml^−1^ for 15 min; supplied by Dr Leslie Loew; Yan et al., 2012), as described previously (Bryant et al., 2015). Line scans were recorded along a t‐tubule, with excitation at 514 nm and emitted fluorescence collected between 518 and 560 nm for Ca^2+^, and between 590 and 700 nm for voltage. Release of Ca^2+^ at the t‐tubule was determined as described previously (Bryant et al., 2015). In brief, the di‐4‐AN(F)EPPTEA fluorescence was used to determine the upstroke of the AP at the t‐tubule. The latency of Ca^2+^ release at each point along the scan was determined from the time between the upstroke of the AP and the time when the Ca^2+^ signal became >5 SD above the prestimulus value. The latency to the time of maximal rate of rise of Ca^2+^ was also determined, and the SD of latencies for each cell was used as a measure of the heterogeneity of release. Spatially averaged (whole‐cell) Ca^2+^ transients were recorded via 1 ms line scans along the length of cells loaded with fluo‐4 AM only, using 488 nm excitation and recording fluorescence between 495 and 650 nm.

### 2.7 Western blot analysis

Samples of myocyte lysates (10 μg protein) were run on 4–15% gradient SDS‐PAGE gels and transferred onto an Immobilon‐P membrane. The blot was probed with antibodies to Cav3 (BD Transduction Laboratories, San Jose, CA, USA; 610420, dilution 1:5000), junctophilin 2 (Jph2, Thermo Fisher Scientific, Waltham, MA, USA; 40‐5300, dilution 1:500), bridging Integrator 1 (Bin1; Santa Cruz Biotechnology Inc., Dallas, TX, U.S.A.; SC‐23918, dilution 1:200) and Gapdh (Sigma; G9545, dilution 1:100,000). Protein bands were visualized and images captured using horseradish peroxidase‐conjugated secondary antibodies (Promega, Madison, WI, USA; W4011, α‐rabbit HRP, dilution 1:10,000 and W4021, α‐mouse HRP, dilution 1:10,000), chemiluminescence and a G:BOX Chemi XT4 imaging system (Syngene, Cambridge, Cambridgshire, UK). The densities of the bands were measured using ImageJ and normalized to Gapdh, with a cross‐check supplied by an internal control for loading.

### 2.8 Statistics

Data are expressed as means ± SD, unless stated, of *N* animals for *in vivo* data and *n*/*N* cells/animals for *in vitro* data, as shown in the figure legends. Data normality was assessed using the Shapiro–Wilk test, and subsequent testing was performed using Student's unpaired *t* test or the Mann–Whitney *U* test; one‐way ANOVA (*post hoc* tests used Bonferroni correction) or the Kruskal–Wallis test. The *I*_Ca_ density–voltage relationship curves were analysed using a repeated‐measures (RM) ANOVA, with voltage and intervention (i.e. DT and C3SD) as factors (Figures 6 and 7); *I*_Ca_ properties elicited by a step depolarization to a single voltage were analysed with two‐way ANOVA; *post hoc* tests used the Bonferroni correction (Figures 6 and 7). The errors in derived variables (specifically, *I*_Ca_ density at the t‐tubule membrane; Figure 6) and the subsequent statistical analysis (Student's unpaired *t* test), were calculated using propagation of errors from the source measurements and thus, to indicate the precision with which the mean value has been estimated, are expressed as SEM. The limit of statistical confidence was taken as *P *< 0.05.

## 3 RESULTS

### 3.1 Severity of response to TAC

**Figure 1:**
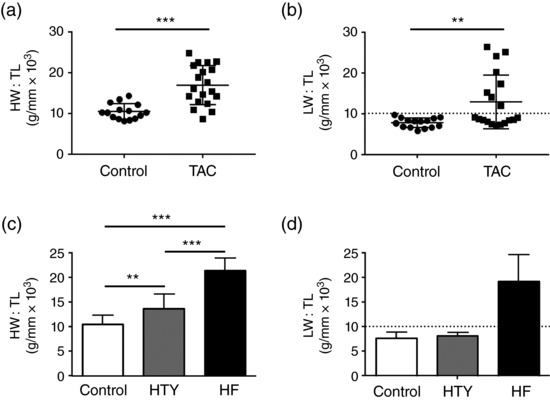
Changes in heart weight and lung weight induced by transverse aortic constriction (TAC). (a, b) Scatter plots with bars showing means ± SD for the ratio of heart weight to tibia length (HW:TL; a) and lung weight to tibia length (LW:TL; b) of control (*N* = 15) and TAC (*N* = 19) animals. The dashed line in (b) shows the demarcation between hypertrophy (HTY) and heart failure (HF; 10.2 g/mm × 10^3^, mean + 2 SD of control). (c, d) HW:TL (c) and LW:TL (d), separated into control (*N* = 15), HTY (*N* = 11) and HF (*N* = 8) groups (as described in the main text). ^**^ *P *<* *0.01, ^***^ *P *<* *0.001; (a) Student's *t* test; (b) Kruskal–Wallis test; (c, d) one‐way ANOVA, Bonferroni‐corrected *post hoc* test

Transverse aortic constriction resulted in cardiac hypertrophy, indicated by a significant increase in the ratio of heart weight to tibia length (HW:TL ratio; in grams per millimetre × 10^3^) from 10.5 ± 1.9 (*N* = 15) in control animals to 17.0 ± 4.8 (*N* = 19) in TAC animals (*P *< 0.001; Figure 1a). Pulmonary congestion, a symptom of congestive heart failure, also increased in TAC animals, indicated by an increase in LW:TL (in grams per millimetre × 10^3^) from 7.8 ± 1.2 (*N* = 15) in control animals to 12.9 ± 6.6 (*N* = 19) after TAC (*P *< 0.01; Figure 1b). The large variability in LW:TL in the TAC group (non‐normal distribution; Shapiro–Wilk *P *< 0.001) and the increase in the coefficient of variation from 15.1 in control animals to 51.3 after TAC (Figure 1b) suggested a graded response to pressure overload. We therefore used LW:TL as an index of heart failure to separate the TAC cohort into two groups: hypertrophy (HTY) and failing (HF), defined as LW:TL < 2 SD and LW:TL > 2 SD above the mean of the control group, respectively (indicated by the dashed line in Figure 1b). Using this definition, HTY mice (*N* = 11) showed cardiac hypertrophy (Figure 1c) but not pulmonary congestion (Figure 1d). In contrast, HF mice (*N* = 8) showed greater hypertrophy (Figure 1c) and pulmonary congestion (Figure 1d).

**Figure 2:**
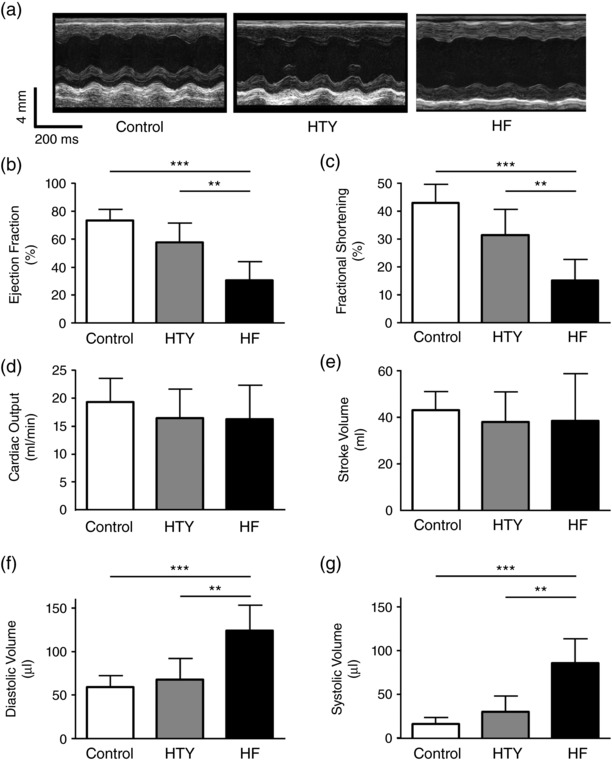
*In vivo* measurement of cardiac function in control, HTY and HF mice. (a) Representative *in vivo* M‐mode echocardiograms from control (left), HTY (middle) and HF (right) mice. (b–f) Mean data showing ejection fraction (b), fractional shortening (c), cardiac output (d), stroke volume (e), diastolic volume (f) and systolic volume (g) in control (*N* = 7), HTY (*N* = 5) and HF (*N* = 7) mice. ^**^ *P *<* *0.01, ^***^ *P *<* *0.001; one‐way ANOVA, Bonferroni‐corrected *post hoc* test

Cardiac function was assessed *in vivo* using M‐mode echocardiography; example records are shown in Figure 2a. These data show that there was a small, statistically non‐significant, decrease in ejection fraction (EF) and fractional shortening (*P *= 0.06) in mice classified as HTY, but that both EF and fractional shortening decreased significantly in HF (Figure 2b,c). Perhaps surprisingly, these changes were not accompanied by significant changes in either cardiac output or stroke volume in either group (Figure 2d,e), although diastolic and systolic volume were significantly larger in HF than in control or HTY mice (Figure 2f,g); heart rate was constant between groups (not shown). Thus, the structural and functional differences between HTY and HF mice shown in Figures 1 and 2 support the idea that increased lung congestion is an appropriate indicator of heart failure (Liao et al., 2002). Echocardiography also showed that the aortic diameter proximal to the stenosis was greater in HF (1.95 ± 0.16 mm, *N* = 7) than in HTY mice (1.75 ± 0.22 mm, *N* = 5) and larger than in control mice (1.66 ± 0.19 mm, *N* = 7; *P *= 0.03, one‐way ANOVA).

### 3.2 Cell size and structure

**Figure 3:**
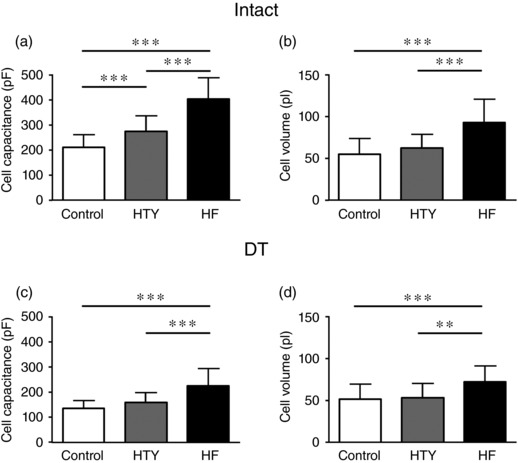
Changes in cell size in HTY and HF. (a, b) Cell capacitance (in picofarads; a) and cell volume (in picolitres; b) measured and calculated, respectively, from intact control (*n*/*N* = 41/10), HTY (*n*/*N* = 22/7) and HF (*n*/*N* = 21/5) myocytes. (c, d) Cell capacitance (in picofarads; c) and cell volume (in picolitres; d) measured and calculated, respectively, from detubulated (DT) control (*n*/*N* = 43/10), HTY (*n*/*N* = 25/7) and HF (*n*/*N* = 21/5) myocytes. ^**^ *P *<* *0.01, ^***^ *P *<* *0.001; one‐way ANOVA, Bonferroni‐corrected *post hoc* test

Analysis of cell morphology revealed that hypertrophy in response to TAC was also graded at the cellular level. In intact myocytes, cell capacitance increased by 30% in HTY and by 91% in HF (Figure 3a). Cell volume, calculated from cell width and length, also showed a graded increase (Figure 3b). In DT myocytes, cell capacitance and cell volume increased with TAC (*P *< 0.001 for both). However, neither cell capacitance nor cell volume increased in HTY cells, but both increased significantly in HF (Figure 3c,d). These data are consistent with cellular hypertrophy increasing with the severity of the cardiac response to pressure overload.

**Figure 4:**
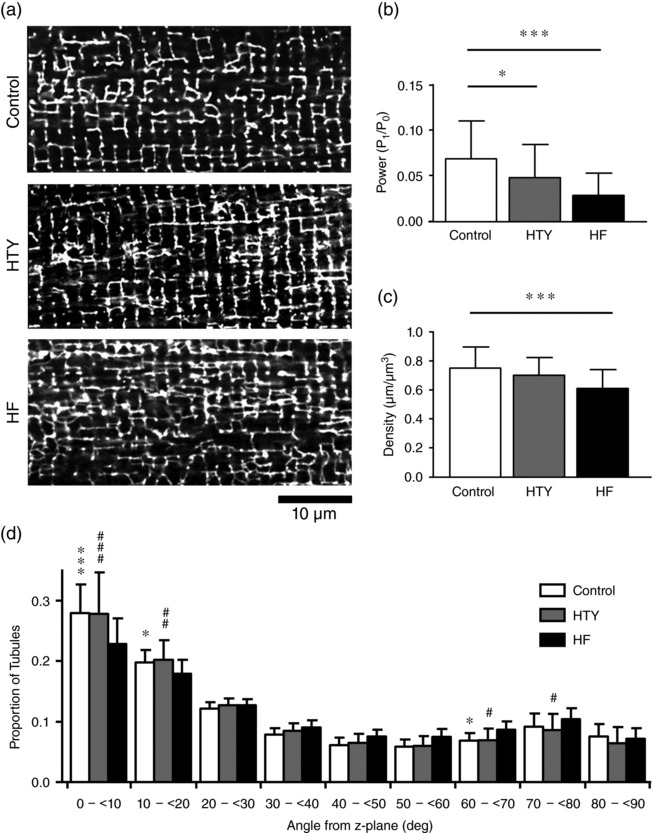
Changes in t‐tubule morphology in HTY and HF. (a) Representative confocal images of control (top), HTY (middle) and HF (bottom) myocytes, stained with di‐8‐ANEPPS. (b) The power of the fast Fourier transformation first harmonic (*P*_1_/*P*_0_). (c) T‐tubule density. (d) T‐tubule orientation (angles in degrees from the z‐disc; data binned between the angles shown on the *x*‐axis) for control (*n*/*N* = 40/8), HTY (*n*/*N* = 26/5) and HF (*n*/*N* = 21/5) myocytes. ^*^ *P* < 0.05 or ^#^ *P* < 0.05, ^**^ *P* < 0.01 or ^##^ *P* < 0.01, ^***^ *P* < 0.001 or ^###^ *P* < 0.001; two‐way ANOVA, Bonferroni‐corrected *post hoc* test. In (d), asterisks indicate a significant difference between control and HF, and hash symbols indicate a significant difference between HTY and HF, at the same angle; there were no significant differences between control and HTY at any angle

Heart failure has previously been shown to be associated with disrupted t‐tubule structure, and representative images of t‐tubules from the control, HTY and HF groups (Figure 4a) appear to show an increasing propensity for discontinuities in the t‐tubule network and an increase in longitudinal or oblique elements (Louch et al., 2006). Fourier analysis confirmed this impression. The amplitude of the first harmonic of t‐tubule labelling (*P*_1_/*P*_0_) was reduced in TAC myocytes (*P *< 0.001) and showed a significant graded decrease (Figure 4b), indicating that t‐tubule regularity is reduced progressively with hypertrophy and failure (Wei et al., 2010). Further analysis revealed that TAC was associated with a graded reduction in t‐tubule density (*P *< 0.001; Figure 4c) and alteration in t‐tubule orientation (Figure 4d), both of which contribute to the decreased t‐tubule regularity. Figure 4d shows that although t‐tubule orientation was not significantly different in the HTY group, cells in the HF group showed a marked decrease in the proportion of transversely oriented tubules, with a modest redistribution to non‐transverse orientations.

**Figure 5:**
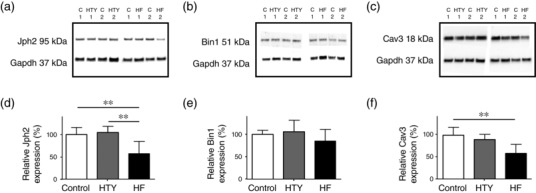
Changes in protein expression after TAC. (a–c) Representative Western blots for Jph2 (a), Bin1 (b) and Cav3 (c) and Gapdh expression in myocyte lysates taken from two control (C1 and C2, replicated on the three blots), two HTY (HTY1 and HTY2) and two HF (HF1 and HF2) hearts. (d–f) Mean data for Jph2 (d), Bin1 (e) and Cav3 (f) protein expression normalized to Gapdh in control, HTY and HF hearts (*N* = 5, 4 and 4 hearts, respectively, each analysed in duplicate). ^**^ *P *<* *0.001, one‐way ANOVA, Bonferroni‐corrected *post hoc* test

To investigate whether the graded changes in t‐tubule structure observed in HTY and HF myocytes could be attributable to graded changes in expression of Jph2, Bin1 and/or Cav3, all of which have been implicated in t‐tubule formation and maintenance (Caldwell et al., 2014; Ziman, Gómez‐Viquez, Bloch, & Lederer, 2010) and have been reported to change in HF (Frisk et al., 2016; Reynolds et al., 2016), we investigated the expression of these proteins in each group. Representative Western blots and mean data are shown in Figure 5. Jph2 expression decreased after TAC (*P *< 0.01), and although it was unaltered in HTY, it was significantly lower in HF (Figure 5d). In contrast, Bin1 expression did not change significantly in either HTY or HF (Figure 5e). However, Cav3 showed a graded decrease in expression with the severity of response to TAC (*P *< 0.01; Figure 5f). Thus, the morphological changes observed during HTY occurred with no significant change in Jph2, Bin1 or Cav3 expression, whereas in HF there was a significant decrease in Jph2 and Cav3.

### 3.3 Distribution and regulation of *I*_Ca_

**Figure 6:**
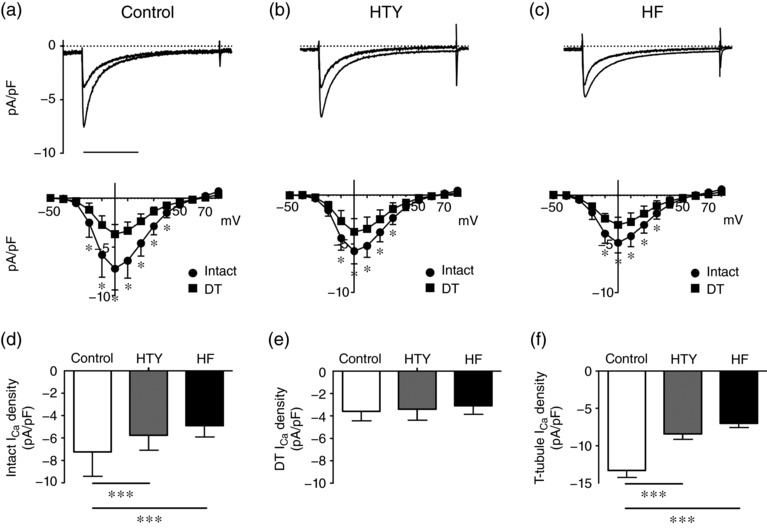
Changes in L‐type Ca^2+^ current (*I*_Ca_) associated with TAC. (a–c) Top panels are representative records of *I*_Ca_ from intact and detubulated (DT) control (a), HTY (b) and HF (c) myocytes. Scale bar represents 200 ms. The corresponding mean *I*_Ca_ density–voltage relationships are shown below for intact (circles) control (*n*/*N* = 41/10), HTY (*n*/*N* = 22/7) and HF (*n*/*N* = 21/5) myocytes and detubulated (squares) control (*n*/*N* = 43/10), HTY (*n*/*N* = 25/7 and HF (*n*/*N* = 21/5) myocytes. (d) Mean *I*_Ca_ density at 0 mV in intact control, HTY and HF myocytes. (e) Mean *I*_Ca_ density at 0 mV in DT control, HTY and HF myocytes. (f) Mean ± SEM *I*_Ca_ density (in picoamperes per picofarad) calculated at the t‐tubule membrane (see Methods). (a–c) ^*^ *P *<* *0.001, two‐way repeated‐measures ANOVA, Bonferroni‐corrected *post hoc* test. (d, e) ^***^ *P *<* *0.001, two‐way ANOVA, Bonferroni‐corrected *post hoc* test. (f) ^***^ *P *<* *0.001, one‐way ANOVA, Bonferroni‐corrected *post hoc* test

To determine the distribution of *I*_Ca_ between the surface and t‐tubular membranes, *I*_Ca_ was measured in intact and DT myocytes. Figure 6 shows records of *I*_Ca_ recorded at 0 mV from representative intact and DT control (Figure 6a), HTY (Figure 6b) and HF (Figure 6c) myocytes, with the corresponding mean *I–V* relationships below. Detubulation decreased *I*_Ca_ density in control, HTY and HF myocytes (all *P *< 0.001). In intact cells, *I*_Ca_ density (at 0 mV) showed a graded decrease with TAC (Figure 6d). In contrast, *I*_Ca_ density in DT myocytes was not significantly different between the three groups (Figure 6e), showing that *I*_Ca_ density at the surface membrane is not changed in HTY or HF, and thus that the reduction in *I*_Ca_ density in intact myocytes must be at the t‐tubule membrane. Calculation of t‐tubular *I*_Ca_ density supports this idea. Figure 6f shows that *I*_Ca_ density at the t‐tubule membrane decreases in a graded manner with severity of response to TAC; *I*_Ca_ density was reduced in HTY (*P *< 0.001 *versus* control) and further reduced in HF (*P *< 0.001 *versus* control).

**Figure 7:**
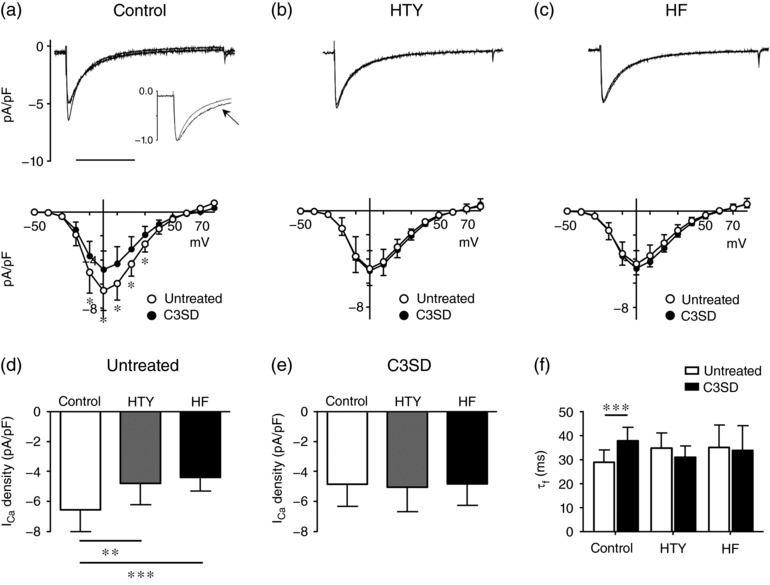
Regulation of *I*_Ca_ by caveolin 3 in TAC. (a–c) Top panels are representative records of *I*_Ca_ recorded from intact control (a), HTY (b) and HF (c) myocytes with or without pre‐incubation with C3SD. The inset in (a) shows normalized *I*_Ca_ to illustrate the slowing of inactivation of *I*_Ca_ in the C3SD‐incubated myocyte (denoted by the arrow). Scale bar represents 200 ms. The corresponding mean *I*_Ca_ density–voltage relationships are shown below. Open circles show *I*_Ca_ without C3SD pretreatment (control *n*/*N* = 16/5, HTY *n*/*N* = 14/4 and HF *n*/*N* = 19/5), and filled circles show *I*_Ca_ after incubation with C3SD (control *n*/*N* = 17/5, HTY *n*/*N* = 12/4 and HF *n*/*N* = 15/5). (d) Mean *I*_Ca_ density at 0 mV recorded in untreated intact control, HTY and HF myocytes. (e) Mean *I*_Ca_ density at 0 mV recorded in intact control, HTY and HF myocytes pre‐incubated with C3SD. (f) The fast component of *I*_Ca_ inactivation (τ_f_, in milliseconds, at 0 mV) in untreated (open bars) and C3SD‐treated (filled bars) control, HTY and HF myocytes. (a–c) ^*^ *P *<* *0.001; two‐way repeated‐measures ANOVA, Bonferroni‐corrected *post hoc* test. (d–f) ^**^ *P *<* *0.01, ^***^ *P *<* *0.001; two‐way ANOVA, Bonferroni‐corrected *post hoc* test

Bin1 and Cav3 have been implicated in localization of L‐type Ca^2+^ channels and *I*_Ca_ to the t‐tubules (Bryant et al., 2014; Hong et al., 2010). Given that Cav3, but not Bin1, decreased significantly after TAC, we investigated whether the observed decrease in Cav3 expression played a role in the decrease of *I*_Ca_ density by investigating the effect of C3SD peptide (see Methods) on *I*_Ca_. Figure 7a–c shows representative records of *I*_Ca_ (top) and mean *I*_Ca_ density–voltage curves (bottom) in control, HTY and HF myocytes that were untreated or had been pretreated with C3SD. In control myocytes, C3SD significantly decreased *I*_Ca_ density (*P *< 0.01), suggesting significant Cav3‐dependent stimulation of *I*_Ca_. However, *I*_Ca_ density was not significantly altered by C3SD in either HTY (*P* = 0.7) or HF (*P* = 0.3) myocytes, suggesting that Cav3‐dependent augmentation of *I*_Ca_ is lost after TAC. In support of this idea, although *I*_Ca_ density was significantly larger in untreated control than in untreated HTY and HF myocytes (Figure 7d), after treatment with C3SD the *I*_Ca_ density was not significantly different in control, HTY and HF myocytes, and was similar to the *I*_Ca_ density observed in untreated HF myocytes (Figure 7e). These data suggest that the loss of Cav3‐dependent augmentation might account for the reduction in *I*_Ca_ density in intact myocytes in HF. Likewise, both τ_f_ (Figure 7f) and τ_s_ (data not shown) of *I*_Ca_ were prolonged by C3SD in control myocytes, but neither was changed by C3SD in HTY and HF myocytes. Together, these data suggest loss of Cav3‐dependent regulation of *I*_Ca_ after TAC, consistent with the idea that the decrease in t‐tubular *I*_Ca_ density in HF is attributable, at least in part, to loss of regulation by Cav3.

### 3.4 Calcium release after TAC

**Figure 8:**
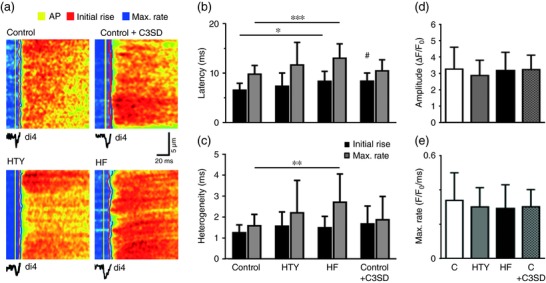
Calcium release after TAC. (a) Transverse line‐scan images of Ca^2+^ transients (field stimulated at 0.2 Hz) and (below) the corresponding spatially averaged di‐4‐AN(F)EPPTEA signal, recorded at a t‐tubule in representative control, HTY and HF myocytes and control myocytes pre‐incubated in C3SD. The yellow, red and blue lines indicate the start time of the t‐tubule action potential, the initial rise of Ca^2+^ and the maximal rate of rise of Ca^2+^, respectively. (b) Mean data showing the latency from the start of the t‐tubule action potential to initial Ca^2+^ release (black bars) and to the maximal rate of rise of Ca (grey bars) in control (*n*/*N* = 43/12), HTY (*n*/*N* = 25/4), HF (*n*/*N* = 12/3) and control + C3SD (*n*/*N* = 24/3) myocytes. (c) Mean data showing the heterogeneity of Ca^2+^ release from the start of the t‐tubule action potential to initial Ca^2+^ release (black bars) and to the maximal rate of rise of Ca^2+^ (grey bars) in control, HTY, HF and control + C3SD myocytes. (d, e) Mean whole‐cell Ca^2+^ transient amplitude (d) and maximal rate of rise (e) in control (*n*/*N* = 53/13), HTY (*n*/*N* = 16/4) and HF (*n*/*N* = 14/3) myocytes and control myocytes with C3SD pretreatment (*n*/*N* = 25/3). ^*^ *P *<* *0.05, ^**^ *P *<* *0.01, ^***^ *P *<* *0.001; Kruskall–Wallis test, Dunn's multiple comparisons test. ^#^ *P* < 0.05, ^##^ *P* < 0.01; Mann–Whitney *U* test between control and control + C3SD

To determine whether the observed changes in *I*_Ca_ were associated with altered Ca^2+^ release at the t‐tubules, the latency between membrane depolarization and subsequent Ca^2+^ release at a t‐tubule was examined as described previously (Bryant et al., 2015). Figure 8a shows transverse line‐scan images of electrically stimulated Ca^2+^ transients and the corresponding spatially averaged di‐4‐AN(F)EPPTEA (membrane potential) signal recorded at a single t‐tubule in representative control, HTY and HF myocytes. Figure 8b shows that the latency of Ca^2+^ release, measured from the start of the AP upstroke to the start of the rise of Ca^2+^ (filled bars) and to the maximal rate of rise of Ca^2+^ (grey bars), became significantly longer in HF, but not HTY, indicating an increase in latency with severity of response to TAC. Figure 8c shows that the heterogeneity of the maximal rate of rise of Ca^2+^ also increased significantly in HF but not in HTY. These changes could not be ascribed to changes in SR Ca^2+^ content, measured using 10 mm caffeine, which did not change significantly, even in HF [*F*/*F*_0_; control 4.2 ± 2.1 (*n*/*N* = 12/4), HTY 6.2 ± 3.6 (*n*/*N* = 7/4) and HF 3.5 ± 1.0 (*n*/*N* = 10/5), *P *= 0.06].

To investigate whether the loss of Cav3‐dependent stimulation of *I*_Ca_ observed after TAC could account for these changes in Ca^2+^ release, we investigated the effect of inhibiting this stimulation using C3SD on Ca^2+^ release in control myocytes. Figure 8a shows a representative transverse line‐scan image of the effect of C3SD on local Ca^2+^ release. Figure 8b,c presents mean data showing that C3SD significantly increased the latency of the initial rise of Ca^2+^. To determine the effect of these local changes on the rise of Ca^2+^ in the whole cell, we recorded the early phase of spatially averaged Ca^2+^ transients. Figure 8d,e shows mean whole‐cell Ca^2+^ transient amplitude (Figure 8d) and maximal rate of rise (Figure 8e) in control, HTY and HF myocytes, and from C3SD‐treated myocytes. These data show that neither Ca^2+^ transient amplitude nor maximal rate of rise was altered after TAC, nor by C3SD.

Thus, in the mouse, pressure overload is associated with a decrease in t‐tubular *I*_Ca_ density, which is graded with the severity of the response to TAC. This graded decrease in t‐tubular *I*_Ca_ density is associated with graded impairment of Ca^2+^ release at the t‐tubule and accompanied by physical disruption of the t‐tubule network. These changes will decrease the synchronization of local Ca^2+^ releases within the cell, which will contribute to the graded decrease in ejection fraction observed *in vivo*, although it has little effect on the rise of the spatially averaged Ca^2+^ transient.

## 4 DISCUSSION

The present *post hoc* analysis of the response to TAC shows graded changes in cardiac growth and function in response to TAC‐induced pressure overload, which are paralleled by graded disruption of t‐tubule network structure and t‐tubular *I*_Ca_ density, and impairment of CICR and SR Ca^2+^ release. These data provide the first demonstration of loss of t‐tubular *I*_Ca_ after TAC in mice and support the suggestion (Hong & Shaw, 2017) that graded changes in cardiac function reflect graded changes in cell, particularly t‐tubule, structure and function. These changes are accompanied by a loss of Cav3‐dependent regulation of *I*_Ca_ and a decrease in Cav3 and Jph2 protein expression in HF. As there is no change in *I*_Ca_ density at the cell surface after TAC, and Cav3‐dependent stimulation of *I*_Ca_ occurs predominantly at t‐tubules (Bryant et al., 2014), loss of such stimulation might underlie the decrease in t‐tubular *I*_Ca_ density.

### 4.1 Classification of cardiac hypertrophy and failure

Transverse aortic constriction is widely used in mice as an experimental model of pressure overload‐induced left ventricular hypertrophy and heart failure. However, the phenotype of this model can vary with the strain of mouse and the severity of stenosis (Barrick, Rojas, Schoonhoven, Smyth, & Threadgill, 2007; Garcia‐Menendez, Karamanlidis, Kolwicz, & Tian, 2013; Mohammed et al., 2012), even within a single strain, with the same constriction and surgeon, as in the present study. The observation that the aortic diameter proximal to the constriction was largest in the HF animals and smallest in control animals suggests that differences in the severity of the constriction and thus increase in pressure might underlie the differing responses, because even small differences in constriction can result in large changes in resistance, which varies with the fourth power of the radius (Sutera & Skalak, 1993).

We therefore grouped mice according to their LW:TL ratio, and an LW:TL of 2 SD above the mean LW:TL of the control group was taken to indicate congestive heart failure. Mice that showed an LW:TL ratio below the threshold (Figure 1b) were classified as having cardiac hypertrophy but without being in overt heart failure. Other measures of pathology, including ejection fraction and fractional shortening (Figure 2), HW:BW, LW:BW, cell size and changes in t‐tubular organization (Figures 3 and 4), also showed gradation between the control, HTY and HF groups, supporting this classification criterion. Despite these changes, stroke volume and cardiac output were maintained, although the mechanism appeared to be different in HTY and HF animals. The lack of change in HTY mice appeared to be because there was little change in either contraction or heart size (Figures 2b,c,f,g). In contrast, in HF, although fractional shortening and ejection fraction decreased, the heart was larger, so that cardiac output was maintained by ejecting a smaller fraction of a larger diastolic volume (i.e. a dilated cardiomyopathy phenotype).

### 4.2 T‐tubule structure after TAC

Examination and analysis of 3D t‐tubule structure in myocytes from control, HTY and HF mice showed that t‐tubule network disruption is graded with the severity of response to TAC (Figure 4), consistent with 2D Fourier analysis of ventricular tissue from rats after TAC (Wei et al., 2010). However, Fourier analysis does not show the changes in the complex architecture of the t‐tubule network that underlie the changes in regularity. To overcome this, t‐tubule density and orientation, both of which are strongly linked to cardiac contraction (Crossman et al., 2015), were measured in 3D. The t‐tubule density showed a graded reduction with severity of response to TAC (Figure 4c), and there was a change in the orientation of the remaining tubules in the HF group (Figure 4d). These data show that cellular hypertrophy and decreased t‐tubule density occur in response to pressure overload and might precede the development of overt heart failure.

The mechanisms underlying t‐tubule disruption are unknown, although t‐tubules are known to be labile (Balijepalli et al., 2003; Brette & Orchard, 2003; Crossman et al., 2011; Forbes, Hawkey, & Sperelakis, 1984; He et al., 2001; Louch et al., 2004; Ziman et al., 2010). A number of proteins have been implicated in the formation and maintenance of t‐tubules, including Bin1 (Caldwell et al., 2014; Hong et al., 2010), Jph2 (Reynolds et al., 2013) and Cav3 (Parton, Way, Zorzi, & Stang, 1997; Ziman et al., 2010). In the present study, Jph2 and Cav3 expression decreased significantly during HF but not HTY; this is discussed further in section 4.4. These changes in expression, and thus t‐tubule structure, might result from the changes in wall stress that occur after TAC and cardiac remodelling (Frisk et al., 2016; Ibrahim et al., 2010; Louch et al., 2006).

### 4.3 *I*_Ca_ and Ca^2+^ release after TAC

Although most studies suggest that *I*_Ca_ density is unaltered in HF (Benitah et al., 2002; Kamp & He, 2002), recent work in failing rat heart cells has shown a decrease in t‐tubular *I*_Ca_ that is offset by an increase in *I*_Ca_ at the cell surface (Bryant et al., 2015) as implied by earlier work on human heart (Schroder et al., 1998). In the present study, there was a graded decrease in t‐tubular *I*_Ca_ density with increased severity of response to TAC (Figure 6e), although *I*_Ca_ density at the surface membrane was unchanged; therefore, the whole‐cell *I*_Ca_ density decreased. However, the disruption of t‐tubule structure and decreased *I*_Ca_ density in the remaining t‐tubules observed after TAC, both of which will impair Ca^2+^ release, appear to be consistent between species and aetiologies.

To investigate the consequences of the graded decrease in t‐tubular *I*_Ca_ on E–C coupling, we measured Ca^2+^ release at single t‐tubules. There was a small but non‐significant increase in latency and heterogeneity between control and HTY (Figure 8), consistent with near‐normal *in vivo* cardiac function in HTY mice, whereas latency, heterogeneity and *in vivo* function were all impaired in HF mice, although the relatively small fractional increases in latency compared with the large fractional decreases in t‐tubular *I*_Ca_ density (Figure 6e) suggest that the gain of E–C coupling in mice is high; therefore, in normal conditions, *I*_Ca_ density is more than sufficient to activate CICR maximally (Cannell, Berlin, & Lederer, 1987). Given that the intersection with the surface membrane was excluded from measurements of local Ca^2+^ release, these data suggest that latency (and thus, by implication, *I*_Ca_), changes along the t‐tubule, as shown in Figure 8a. We have shown previously that inhibition of *I*_Ca_ using a Ca^2+^ channel blocker causes similar impairment of Ca^2+^ release (Bryant et al., 2015). The use of C3SD in control myocytes to mimic the loss of Cav3‐dependent stimulation of *I*_Ca_ observed after TAC resulted in a similar decrease in *I*_Ca_ (30%) and increase in latency (27%) as observed in HF myocytes (33 and 26%, respectively). Thus, loss of Cav3‐dependent stimulation of *I*_Ca_ is sufficient to account for the decrease in *I*_Ca_ and increase in latency observed in HF.

The increase in latency and heterogeneity of Ca^2+^ release observed in HF will contribute to the increased heterogeneity of Ca^2+^ release reported previously and ascribed predominantly to disruption of t‐tubule structure (Louch et al., 2004, 2006). However, TAC had little effect on the amplitude of the spatially averaged Ca^2+^ transient, consistent with the lack of effect on SR Ca^2+^ content, which has previously been reported to increase, not change or decrease in hypertrophy and HF (Bers, 2006; Gómez et al., 1997b; Ibrahim et al., 2012; Mork et al., 2007). Perhaps surprisingly, given the increase in latency and heterogeneity of local Ca^2+^ release, the maximal rate of rise of the whole‐cell Ca^2+^ transient was not significantly altered by TAC or by pretreatment with C3SD peptide, suggesting that other factors dominate the rising phase of the Ca^2+^ transient. The relationship with the *in vivo* data is complex, both because of the presence of neurohumoral influences *in vivo* and because, although ejection fraction and fractional shortening decreased, this was in the presence of increased heart size. Given that wall tension will increase with dilatation, the Law of Laplace shows that a reduced ejection fraction does not necessarily imply reduced contractility.

It appears, however, that impaired Ca^2+^ release in HF is attributable, in part, to disruption of t‐tubule structure and decreased *I*_Ca_ density in the remaining t‐tubules.

### 4.4 Caveolin 3 levels and regulation of *I*_Ca_ after TAC

The observation that incubation of myocytes with C3SD peptide reduced *I*_Ca_ density and increased τ_f_ and τ_s_ in control cells, but not in HTY or HF myocytes (Figure 7), suggests a constitutive Cav3‐dependent increase in *I*_Ca_ density and inactivation in control myocytes, which is lost after TAC. The reason for this is unknown; however, *I*_Ca_ has previously been shown to be tonically stimulated by protein kinase A (Bracken, ElKadri, Hart, & Hussain, 2006; Bryant et al., 2014; Hussain, Drago, Bhogal, Colyer, & Orchard, 1999). Given that the binding partners of Cav3 include proteins that regulate Ca^2+^ channel phosphorylation (Balijepalli, Foell, Hall, Hell, & Kamp, 2006), and disruption of Cav3 binding using C3SD inhibits such stimulation (Bryant et al., 2014), it appears possible that Cav3 binding to its partners is disrupted after TAC. Previous work has also shown that Cav3‐dependent stimulation of *I*_Ca_ occurs predominantly at t‐tubules (Bryant et al., 2014).

One possible explanation for a loss of Cav3 binding after TAC is a decrease in Cav3 expression. Jph2, Bin1 and Cav3 have been implicated in the development and maintenance of t‐tubules and in the regulation of t‐tubular *I*_Ca_ density (Bryant et al., 2014; Hong et al., 2010). In the present study, there was no change of Bin1 expression in HTY or HF; however, Jph2 and Cav3 expression decreased significantly in HF but not in HTY mice (Figure 5). Thus, the loss of Cav3‐dependent stimulation and significant decrease in t‐tubular *I*_Ca_ density in HTY myocytes occurred with no significant changes in Cav3 expression (Figure 5), whereas in HF myocytes the decrease in t‐tubular *I*_Ca_ density was accompanied by a significant decrease in Cav3 expression. Given the role of Jph2, Bin1 and Cav3 in determining t‐tubule structure and *I*_Ca_ localization (Bryant et al., 2014, 2015; Caldwell et al., 2014; Hong et al., 2010; Parton et al., 1997; Reynolds et al., 2013; Ziman et al., 2010), it appears possible that changes in protein trafficking precede decreased expression, which could explain why a decrease in t‐tubular *I*_Ca_ density and altered t‐tubule structure precede marked decreases in protein expression, which appear to be associated with the HF rather than the HTY phenotype (Reynolds et al., 2016), although it remains possible that another mechanism, as yet unknown, inhibits Cav3‐dependent stimulation of *I*_Ca_.

## COMPETING INTERESTS

None declared.

## AUTHOR CONTRIBUTIONS

All laboratory experiments were performed at the University of Bristol. S.M.B., C.H.T.K., A.F.J., M.B.C. and C.H.O. conceived the study and designed the project methods. S.M.B., C.H.T.K., J.J.W. and H.C.G. contributed to acquisition, analysis or interpretation of data. All authors contributed to the drafting of the manuscript and its revision. All authors approved the final version of the manuscript and agree to be accountable for all aspects of the work in ensuring that questions related to the accuracy or integrity of any part of the work are appropriately investigated and resolved. All persons designated as authors qualify for authorship, and all those who qualify for authorship are listed.
